# Silymarin and diabetic nephropathy

**DOI:** 10.12861/jrip.2012.02

**Published:** 2012-01-01

**Authors:** Mahmoud Rafieian-Kopaie, Hamid Nasri

**Affiliations:** ^1^Medical Plants Research Center, Shahrekord University of Medical Sciences, Shahrekord, Iran; ^2^Department of Nephrology, Division of Nephropathology, Isfahan University of Medical Sciences, Isfahan, Iran

**Keywords:** Silymarin, Diabetic nephropathy, Kidney, *Silybum marianum*

## Abstract

Nephropathy is one of the most important complications of diabetes mellitus and drug induced toxicity. Nephrotoxicity is mostly related to oxidative stress and nowadays much attention has been made towards the possible kidney protective properties of medicinal plants. Studies revealed, silymarin is useful for diabetic nephropathy. The combination of metformin, silymarin and renin-angiotensin system inhibitors or angiotensin receptor blockers may have additive kidney protective property to prevent or slowing the progression of diabetic nephropathy.

**Implication for health policy/practice/research/medical:**

Nephropathy is one of the most important complications of diabetes mellitus and drug induced toxicity. Nephrotoxicity is mostly related to oxidative stress and nowadays much attention has been made towards the possible kidney protective properties of medicinal plants. Studies revealed, silymarin is useful for diabetic nephropathy. The combination of metformin, silymarin and renin-angiotensin system inhibitors or angiotensin receptor blockers may have additive kidney protective property to prevent or slowing the progression of diabetic nephropathy.

## Introduction

About half the medicinal preparations have been developed during the last couple decades and it is perhaps not widely known that botanical-derived products are a major source of new molecules which are used as therapeutic agents. *Silybum marianum* (milk thistle) a medical plant from Asteraceae family and native to the Middle East, Mediterranean regions of Europe and north Africa (1).

*Silybum marianum* and its derivatives are among the commonly used products in the United States and comprise nearly 4% of natural products. The extract of *Silybum marianum* seeds consists of 80% an active flavonoid-lignan (flavonolignan), called silymarin, with antioxidant activity and regenerative properties (1,2). The liver-protective effects of *Silybum marianum* were known and written about in ancient times. Clinical use for a variety of liver disorders, such as hepatitis, has prospered throughout many parts of the world. The active ingredients of the silymarin have not been clearly known, however, the silymarin products on the market are mostly a few flavonoids and a mixture of at least 7 major isomeric flavonolignans including isosilybin B, silybin A, silybin B, silychristin, isosilychristin, isosilybin A, silydianin and one flavonoid (taxifolin) (1-3). Most of the clinical trials with *Silybum marianum* is in the context of treating liver disorders however, it has beneficial effects on a wide variety of disorders, including lowering cholesterol serum levels, reducing insulin resistance in patients with type 2 diabetes, reducing the growth of cancer cells in breast, cervical, and prostate cancers. The workers taking the milk thistle extract showed significant improvement in liver function tests (ALT and AST) and platelet counts vs. the placebo group. The efficacy of silymarin in preventing drug-induced liver damage in patients taking long-term psychotropic drugs has been investigated which have shown promising effects (2-5). Silymarin prevents or slows toxins from entering the liver cells. Hence, the toxins are excreted through the kidneys before they can cause liver damage. The most dramatic example of this is silymarin’s ability to inhibit poisons from the death cap. Amanita phalloides (mushroom) is one of these most notorious liver toxins known to humans. The death rate in emergency rooms from Amanita poisoning is usually 30 to 40 percent (2-6). It acts in part in a similar fashion to detoxify acetaminophen, alcohol and some heavy metals. Silymarin can reduce its toxicity. Silymarin’s protective effect is due to the flavonoid complex silybin, which acts as a potent antioxidant, neutralizing harmful free radicals that result from normal metabolic processes and from the breakdown of toxic substances. At least 10 times as powerful as vitamin E, silymarin also helps increase levels of two additional antioxidants, glutathione and superoxide dismutase (SOD). Surprisingly, other than protective activity, silymarin can help the liver in repairing injured cells and generating new ones, by stimulating protein synthesis through the enzyme RNA polymerase I. Silymarin's regenerative ability has been reported for treating serious conditions such as toxic fatty deposits in the liver, cirrhosis and chronic hepatitis (1-6).

Specific drugs to prevent or treat nephropathy are lacking in the field of conventional drugs. Thus, introduction of botanical products for prevention or treatments of this disorder, particularly diabetic nephropathy, is valuable. Recent evidence suggests that silymarin may be just as important for kidney health as for liver. Silymarin concentrates in kidney cells, where it aids in repairing and regeneration by increasing protein and nucleic acid synthesis. One study suggested that it increased cell replication by 25 to 30 percent which were related to silybin and silychristin, two important components of silymarin. Studies have shown the beneficial effects of silymarin in diabetic nephropathy (4-10). The results of published studies show that silymarin might be effective for prevention of nephropathy-induced premature death in diabetic patients. In this regards a trial conducted on 60 diabetic patients with urinary albumin excretion >300 mg/d to assess a primary end point of absolute change in urine albumin-creatinine ratio explored that the silymarin-treated group had at least a 50% decrease in urine albumin-creatinine ratio after 3 months of treatment. Secondary end points reviled patterns in changes of a panel of some markers that might be related to oxidative stress, inflammation and fibrosis. Silymarin has also been associated with a trend towards reducing the liver damaging effects of chemotherapy in a randomized double-blind placebo controlled study of 50 children (3-10).

The potential efficacy of silymarin in the treatment of diabetic nephropathy has been shown in a few studies. It has also been found to be effective in reducing proteinuria in type 2 diabetes patients with overt nephropathy in a randomized controlled trial. This reduction in proteinuria was related to antioxidant and anti-inflammatory effects of silymarin (4-12). Recently, Fallahzadeh *et al*, investigated the effect of addition of silymarin to renin-angiotensin system inhibitors on proteinuria in type 2 diabetic patients with overt nephropathy. In this study, silymarin could reduce urinary excretion of albumin, TNF-α, and malondialdehyde in patients with diabetic kidney disease (13).

The early observations are noteworthy in that they suggest a potentially novel therapeutic strategy for serious diseases. However, enthusiasm regarding the possible efficacy or safety of silymarin in kidney diseases, especially in diabetic nephropathy should be tempered by the recognition that promising initial reports of botanical products, and of various conventional agents, often are not confirmed. With botanical products, careful characterization of the substance, measurement of bioavailability, and efforts to clarify mechanism of action and accurately identify target entities can all help prevent later disappointments (9-12). Although the clinical trials suggest promising beneficial effects for silymarin as a therapeutic agent in reducing nephropathy, a few specific aspects of this silymarin trial, as well as unique features of plants product trials in general, are worthy of further investigation (8-11).

It should be noted that these compounds are relatively poorly absorbed (20 to 50%) by the gastrointestinal tract. It may suggest that standardization of silymarin or preparation of parenteral or other forms of the drug are important to ensure effective concentrations of the active ingredients. It has also claimed that combining silymarin with phosphatidylcholine increases absorption (7-15).

## Conclusion

Noteworthy to explain that in a recent study, silymarin extract could safely be used together with metformin to increase the antioxidant potency and better renoprotection.

Therefore, combination of metformin, silymarin and renin-angiotensin system inhibitors or angiotensin receptor blockers may have additive kidney protective property beyond controlling the blood sugar of metformin, to prevent or slowing the progression of diabetic nephropathy.

## Authors’ contributions

MRK prepared the primary draft. HN reviewed the manuscript. MRK prepared the final manuscript.

## Conflict of interests

The author declared no competing interests.

## Ethical considerations

Ethical issues (including plagiarism, data fabrication, double publication) have been completely observed by the author.

## Funding/Support

None.
